# Leaflet modification before transcatheter aortic valve implantation in patients at risk for coronary obstruction: the ShortCut study

**DOI:** 10.1093/eurheartj/ehae303

**Published:** 2024-05-15

**Authors:** Danny Dvir, Didier Tchétché, Martin B Leon, Philippe Généreux, Benjamin Seguy, Raj Makkar, Philippe Pibarot, Hemal Gada, Tamim Nazif, David Hildick-Smith, Jörg Kempfert, Nicolas Dumonteil, Axel Unbehaun, Thomas Modine, Brian Whisenant, Christophe Caussin, Lenard Conradi, Thomas Waggoner, Jacob M Mishell, Stanley J Chetcuti, Saibal Kar, Michael J Rinaldi, Molly Szerlip, Ravi K Ramana, Daniel J Blackman, Itsik Ben-Dor, Ran Kornowski, Ron Waksman, Ulrich Gerckens, Paolo Denti, Marian Kukucka, Julien Ternacle, Sabah Skaf, Jan Kovac, Hasan Jilaihawi, Vivek Patel, Rami Jubeh, Mohamed Abdel-Wahab, Susheel Kodali

**Affiliations:** Department of Cardiology, Shaare Zedek Medical Center and Faculty of Medicine, Hebrew University of Jerusalem, P.O. Box 3235, Shmuel Bait 12 Street, Jerusalem 9103102, Israel; Groupe CardioVasculaire Interventionnel, Clinique Pasteur, Toulouse, France; Columbia University Medical Center, NewYork-Presbyterian Hospital, Cardiovascular Research Foundation, New York, NY, USA; Gagnon Cardiovascular Institute at Morristown Medical Center, NJ, USA; CHU de Bordeaux, Bordeaux, France; Cedars-Sinai Medical Center, Smidt Heart Institute, Los Angeles, CA, USA; Department of Cardiology, Quebec Heart and Lung Institute, Laval University, Quebec City, Quebec, Canada; UPMC Harrisburg/Pinnacle Health Cardiovascular Institute, Wormleysburg, PA, USA; Columbia University Medical Center, New York Presbyterian, New York, NY, USA; University Hospital Sussex, Royal Sussex County Hospital, Brighton, UK; Deutsches Herzzentrum der Charité, Department of Cardiothoracic and Vascular Surgery, Berlin, Germany; Charité-Universitätsmedizin Berlin, Germany DZHK (German Center for Cardiovascular Research), Partner Site Berlin, Germany; Groupe CardioVasculaire Interventionnel, Clinique Pasteur, Toulouse, France; Deutsches Herzzentrum der Charité, Department of Cardiothoracic and Vascular Surgery, Berlin, Germany; Charité-Universitätsmedizin Berlin, Germany DZHK (German Center for Cardiovascular Research), Partner Site Berlin, Germany; CHU de Bordeaux, Bordeaux, France; Department of Cardiology, Intermountain Medical Center, Salt Lake City, UT, USA; Institut Mutualiste Montsouris, Paris, France; University Heart & Vascular Center Hamburg, Germany; US Heart & Vascular, Tucson Medical Center, Tucson, AZ, USA; Kaiser Permanente Northern California Structural Heart Program, San Francisco, CA, USA; University of Michigan, Ann Arbor, MI, USA; Los Robles Regional Medical Center, Thousand Oaks, CA, USA; Sanger Heart & Vascular Institute, Charlotte, NC, USA; Baylor Scott & White The Heart Hospital, Plano, TX, USA; Advocate Christ Medical Center Oak Lawn, IL, USA; Heart Care Centers of Illinois, Palos Park, IL, USA; 22Leeds Teaching Hospitals, Leeds, UK; Leeds Teaching Hospitals, Leeds, UK; Section of Interventional Cardiology, MedStar Washington Hospital Center, Washington, DC, USA; Rabin Medical Center, Petah Tikva & Faculty of Medicine, Tel Aviv University, Israel; Section of Interventional Cardiology, MedStar Washington Hospital Center, Washington, DC, USA; Bonn, Germany; Cardiac Surgery Department, San Raffaele University Hospital, Milan, Italy; Deutsches Herzzentrum der Charité, Department of Cardiothoracic and Vascular Surgery, Berlin, Germany; Charité-Universitätsmedizin Berlin, Germany DZHK (German Center for Cardiovascular Research), Partner Site Berlin, Germany; CHU de Bordeaux, Bordeaux, France; Cedars-Sinai Medical Center, Smidt Heart Institute, Los Angeles, CA, USA; University Hospital of Leicester, Leicester, UK; Cedars-Sinai Medical Center, Smidt Heart Institute, Los Angeles, CA, USA; Cedars-Sinai Medical Center, Smidt Heart Institute, Los Angeles, CA, USA; Department of Cardiology, Shaare Zedek Medical Center and Faculty of Medicine, Hebrew University of Jerusalem, P.O. Box 3235, Shmuel Bait 12 Street, Jerusalem 9103102, Israel; Heart Center Leipzig at University of Leipzig, Leipzig, Germany; Columbia University Medical Center, New York Presbyterian, New York, NY, USA

**Keywords:** Transcatheter aortic valve implantation, Valve Academic Research Consortium, Valve in valve, Virtual transcatheter heart valve to coronary artery, Virtual transcatheter heart valve to sinotubular junction

## Abstract

**Background and Aims:**

This trial sought to assess the safety and efficacy of ShortCut, the first dedicated leaflet modification device, prior to transcatheter aortic valve implantation (TAVI) in patients at risk for coronary artery obstruction.

**Methods:**

This pivotal prospective study enrolled patients with failed bioprosthetic aortic valves scheduled to undergo TAVI and were at risk for coronary artery obstruction. The primary safety endpoint was procedure-related mortality or stroke at discharge or 7 days, and the primary efficacy endpoint was per-patient leaflet splitting success. Independent angiographic, echocardiographic, and computed tomography core laboratories assessed all images. Safety events were adjudicated by a clinical events committee and data safety monitoring board.

**Results:**

Sixty eligible patients were treated (77.0 ± 9.6 years, 70% female, 96.7% failed surgical bioprosthetic valves, 63.3% single splitting and 36.7% dual splitting) at 22 clinical sites. Successful leaflet splitting was achieved in all [100%; 95% confidence interval (CI) 94%–100.0%, *P* < .001] patients. Procedure time, including imaging confirmation of leaflet splitting, was 30.6 ± 17.9 min. Freedom from the primary safety endpoint was achieved in 59 [98.3%; 95% CI (91.1%–100%)] patients, with no mortality and one (1.7%) disabling stroke. At 30 days, freedom from coronary obstruction was 95% (95% CI 86.1%–99.0%). Within 90 days, freedom from mortality was 95% [95% CI (86.1%–99.0%)], without any cardiovascular deaths.

**Conclusions:**

Modification of failed bioprosthetic aortic valve leaflets using ShortCut was safe, achieved successful leaflet splitting in all patients, and was associated with favourable clinical outcomes in patients at risk for coronary obstruction undergoing TAVI.

## Structured Graphical Abstract

**Structured Graphical Abstract:**
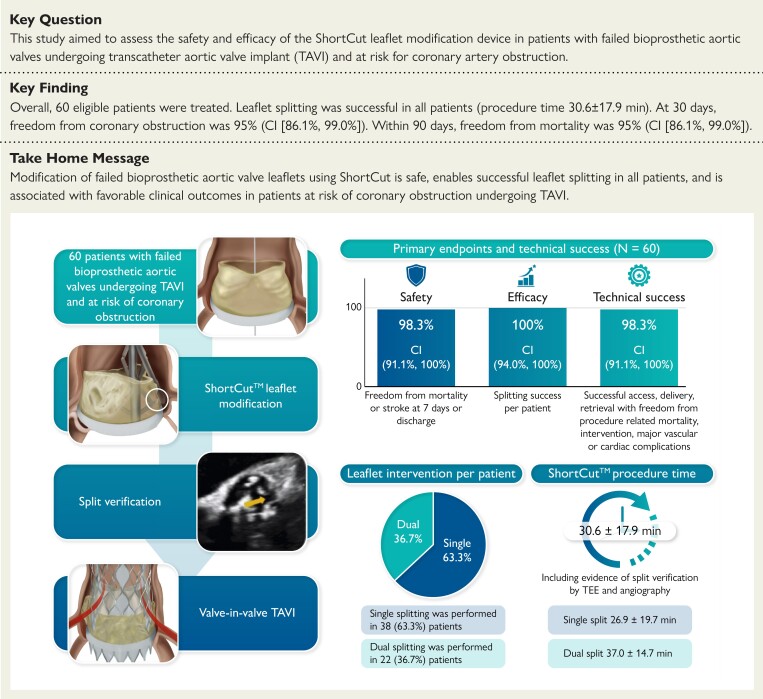
This pivotal prospective, multicentre study presents favourable clinical outcomes of ShortCut, the first dedicated leaflet modification device, prior to transcatheter aortic valve implantation (TAVI) in patients scheduled to undergo TAVI and at risk for coronary artery obstruction.

**See the editorial comment for this article ‘Acute coronary occlusion during valve-in-valve TAVI—a shortcut to successful prevention’, by B.D. Prendergast *et al*., https://doi.org/10.1093/eurheartj/ehae439.**

## Introduction

Transcatheter aortic valve implantation (TAVI) has become the main treatment modality for patients with severe symptomatic aortic valve stenosis and is also commonly used for treatment of failed bioprosthetic valves (valve in valve, ViV).^1–3^ One major concern with the placement of a transcatheter heart valve inside a degenerated bioprosthetic valve is the risk of coronary obstruction, which is associated with a significantly increased risk of mortality.^1,2^ As the need for ViV TAVI procedures is steadily increasing, the incidence of coronary obstruction during TAVI is expected to rise as well. While several transcatheter techniques to lacerate bioprosthetic valve leaflets prior to TAVI placement have been described, their use is complex, time- and resource-consuming, and is associated with an unpredictable success rate; therefore, these techniques have not been adopted widely.^3–7^ Similarly, stenting techniques to maintain sinus flow to the coronary arteries in cases of TAVI-induced coronary obstruction have also been described, but these are considered suboptimal and compromise future coronary access.^8–11^

ShortCut (Pi-Cardia, Rehovot, Israel) is the first dedicated transcatheter device designed to modify valve leaflets by mechanical splitting.^12,13^ Early feasibility experience has shown favourable results but was limited to a small number of patients.^12^ The objective of this study was to assess the safety and efficacy of ShortCut in patients with failed bioprosthetic aortic valves undergoing TAVI who were at risk for coronary obstruction.

## Methods

### Study design

The ShortCut pivotal study is a prospective, investigational device exemption, multicentre study that enrolled patients with failed bioprosthetic aortic valves, scheduled to undergo TAVI, and who were at risk for coronary artery obstruction (see Supplementary data online, *Table S1*). The study was designed by the trial sponsor (Pi-Cardia), the steering committee, with guidance from the US Food and Drug Administration and with approval by the respective institutional ethics committees. Signed informed consent was obtained from all patients before any study-related procedures were conducted (NCT04952909).

### Patients

Patients were eligible for inclusion in the study if they were undergoing transfemoral TAVI for an approved ViV indication and were at risk for TAVI-induced coronary obstruction per local heart team decision. Risk factors for coronary ostium obstruction included high-risk anatomical characteristics, such as low coronary height relative to the annular plane, narrow sinuses of Valsalva, short virtual transcatheter heart valve to coronary artery ostium (VTC) distance, or short virtual transcatheter heart valve to sinotubular junction (VTS) distance. All of these factors have previously been associated with an increased risk of coronary obstruction during ViV.^1,3–6,14^

Both failing surgical and transcatheter bioprosthetic valves were allowed in this study. Details regarding eligibility criteria and frequency distribution of the risk factors are provided in Supplementary data online, *Tables S2* and *S3*. A central screening committee reviewed all patient data, including evaluations of independent computed tomography (CT) and echocardiography core laboratories. The committee assessed the risk for coronary obstruction and made a final decision regarding patient eligibility to participate in the study.

### Pre-procedure assessment and planning

All patients underwent standard pre-TAVI CT assessment to evaluate anatomical suitability for transfemoral ViV TAVI and to identify risk factors for coronary obstruction (see Supplementary data online, *Figure S1*). CT assessments were used to identify viewing projection angles for the left and/or right coronary leaflet splitting. Vascular anatomy was reviewed to ensure suitability for cerebral embolic protection using the Sentinel device (Boston Scientific). Post-dilation or high-pressure balloon post-dilation bioprosthetic frame fracture was planned per operator discretion.^15^ All sites received a mixture of didactic and hands-on simulator training prior to their first case.

### Procedure and ShortCut leaflet splitting

All procedures were guided by fluoroscopy per standard of care. To document leaflet splitting, general anaesthesia and transoesophageal echocardiography (TEE) were used. As no prior data were available on embolic risk in this high-risk population, and to standardize the treated cohort, a cerebral embolic protection device was placed via the right radial artery prior to study device introduction. ShortCut was utilized by physicians trained in its use, without necessitating prior experience for a single leaflet split procedure. After standard transfemoral vascular access was achieved, the ShortCut was introduced via a 16 F sheath. It was recommended that activated clotting time should be maintained above 250 s during the procedure. Transcatheter aortic valve implantation device selection was according to operator discretion.

**Figure 1:**
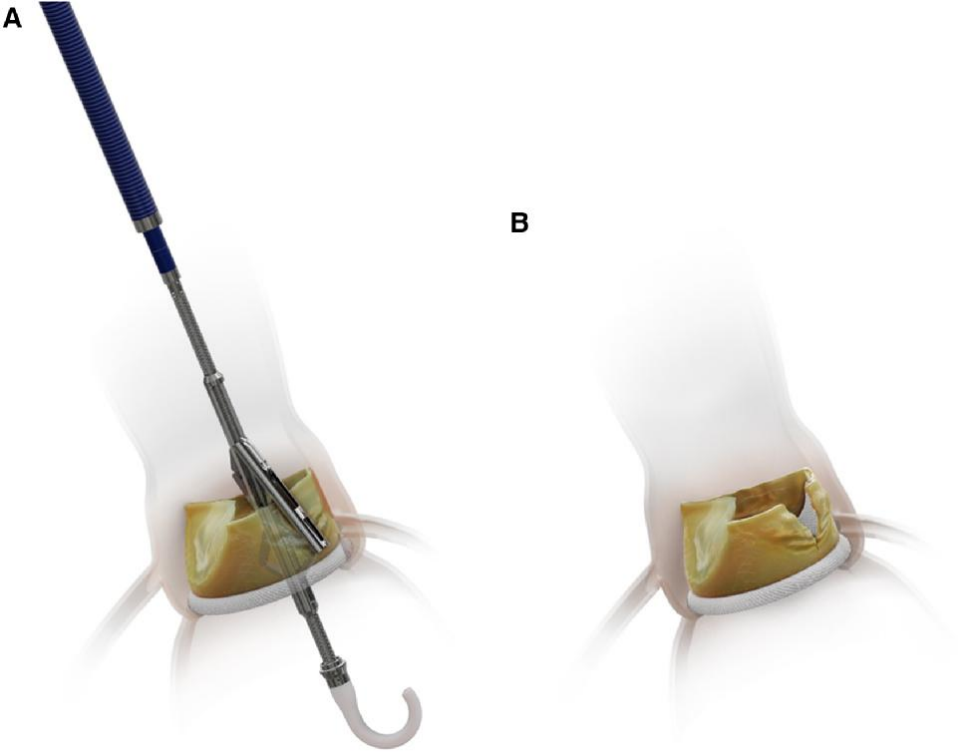
The ShortCut device. (*A*) The ShortCut device positioned at the bottom of the cusp with the splitting element activated. (*B*) Post-ShortCut split of the failed surgical valve

The ShortCut device was used as previously described (*Figure 1*).^12,13^ Briefly, the device is introduced under fluoroscopic guidance over a standard 0.035 inch guidewire and advanced to the ascending aorta. After crossing the aortic valve, the device is unsheathed to automatically open the positioning arm above the valve. A deflection mechanism allows the device to centre in the valve. Pre-determined fluoroscopic views are then used to rotate and advance the positioning arm to accurately and on the base of the target leaflet for split. After confirming positioning arm location, the splitting element is activated to puncture the leaflet base from the ventricular aspect. The catheter is then gently retracted, performing a vertical split of the leaflet upward from base to tip. If required, the positioning arm is then rotated towards a second target leaflet and the splitting procedure is repeated (dual split). Afterwards, the ShortCut device is removed, and TAVI is performed over the same guidewire. Per study protocol, only one splitting attempt per leaflet was allowed. Transoesophageal echocardiography was conducted during the procedure for standardized acquisition of echocardiography images before and after leaflet splitting. Following TAVI, coronary artery patency was assessed using angiography and colour Doppler echocardiography. Echocardiography and angiography acquisitions were evaluated by independent core laboratories using a standardized protocol. ShortCut procedure time was defined as the time from ShortCut catheter insertion to its retrieval from the introducer sheath, including imaging time required to confirm leaflet splitting by TEE and aortography. Post-procedure antiplatelet and/or anticoagulation therapy was prescribed at operator discretion.

### Study endpoints

The primary safety endpoint was ShortCut device- and/or procedure-related mortality and/or stroke at discharge or 7 days post-procedure, whichever occurred first. The primary efficacy endpoint was per-patient leaflet splitting success, determined intra-procedurally by visualization of leaflet split on TEE or increase in aortic regurgitation, assessed by independent echocardiography or angiography core laboratories. For patients in whom dual splitting was planned, per-patient leaflet splitting success was determined based on the splitting success of the first leaflet.

The secondary safety endpoints were defined as all-cause mortality, all-cause stroke, coronary ostium obstruction, myocardial infarction with new evidence of coronary artery obstruction requiring intervention, major vascular complication, cardiac tamponade, acute stage 3–4 kidney injury, and access-related type 3–4 bleeding at 30 days, as defined by Valve Academic Research Consortium-3 (VARC-3) criteria.^16^ Secondary efficacy endpoints included: intraprocedural per-leaflet splitting success, 30-day freedom from intervened leaflet-related coronary artery obstruction and intervened leaflet-related coronary artery intervention. Technical success was assessed on exit from the procedure room, and was a composite of successful access, delivery and retrieval of the ShortCut device, freedom from ShortCut device- or procedure-related mortality, freedom from ShortCut device- or procedure-related surgery, intervention, major vascular complications, and cardiac structural complications.

All patients underwent transthoracic echocardiography, 12-lead electrocardiogram (ECG), and a neurological evaluation including modified Rankin score and NIH Stroke Scale at baseline and at discharge or 7 days after the procedure, the earlier of the two. A physical examination, 12-lead ECG, and neurological evaluation were conducted 30 days after the procedure. At 90 days post-procedure, patient overall clinical status, adverse events, and concomitant medications were evaluated. Safety events were adjudicated throughout the study according to VARC-3 criteria by a clinical events committee that comprised of two independent physicians.^16^ In addition, ShortCut device and procedure safety aspects were periodically reviewed by a data safety monitoring board.

### Statistical analysis

The primary efficacy analysis set included patients in whom split with the ShortCut device was attempted and were determined to have adequate imaging to demonstrate evidence of a split according to either echocardiography or angiography core lab assessments. Inadequate imaging included technical limitations which were unrelated to the ShortCut device or procedure success, such as missing images or poor image quality. The safety analysis set included all patients who reached the point of index procedure (i.e. the ShortCut device was introduced through the introducer sheath). The study was statistically powered to achieve significance for the primary efficacy endpoint with a performance goal of 75% of patients with a successful split, using exact binomial test. Assuming a 90% rate of patients with a successful split, a sample size of 60 patients would provide at least 80% power to reject the primary effectiveness null hypothesis. Continuous parameters are presented as mean ± standard deviation; categorical parameters are presented as count and percentages, with 95% two-sided confidence intervals (CIs) based on Clopper–Pearson score. All statistical analyses were carried out using SAS version 9.4 (SAS Institute, Cary, NC, USA) under Windows 2016 Terminal.

## Results

### Baseline characteristics

**Figure 2:**
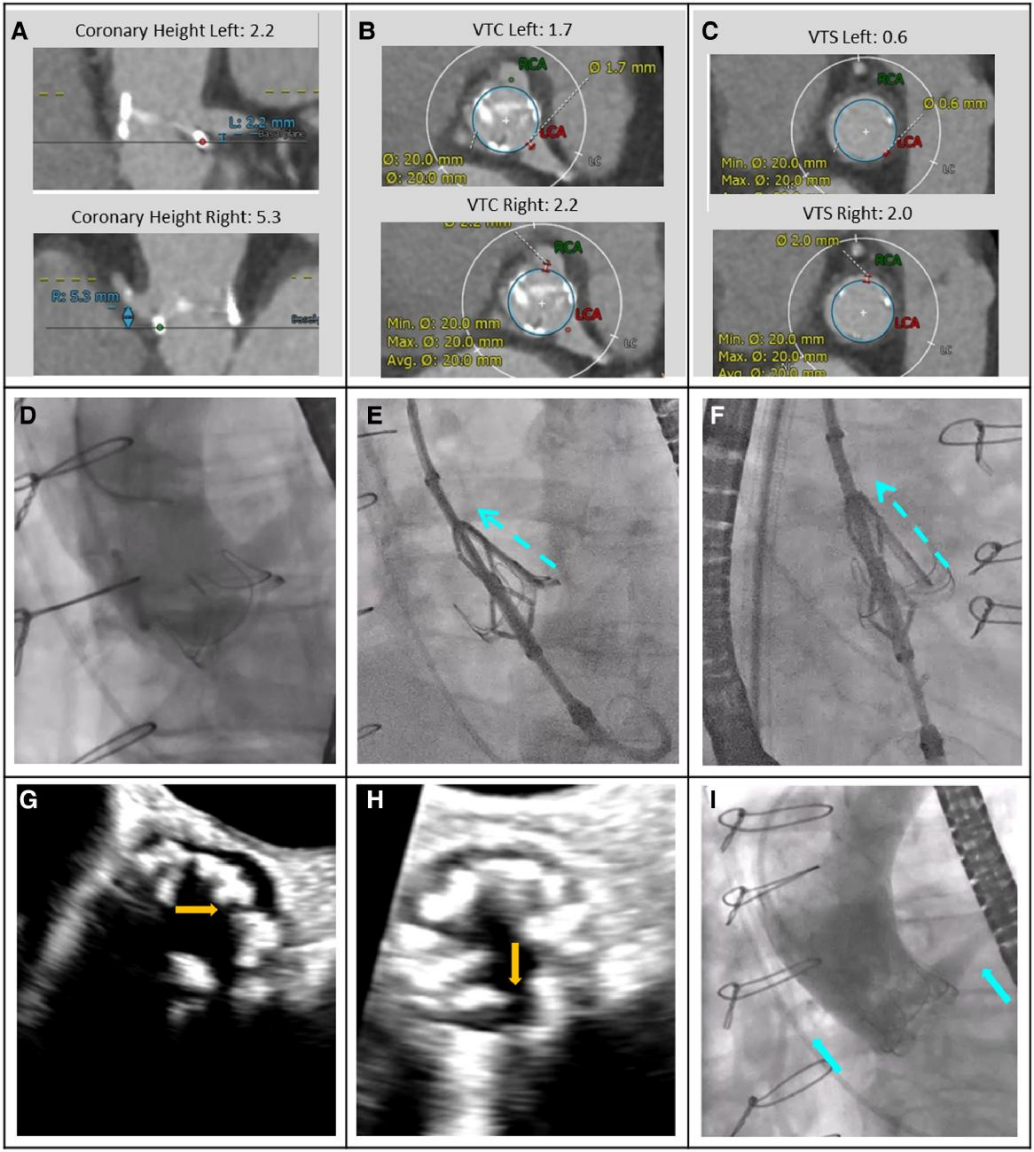
A ShortCut procedure in a patient with a failed bioprosthetic surgical valve. A 76-year-old female with a failed Magna Ease 21 mm surgical bioprosthetic aortic valve (Edwards Lifesciences) at high risk for double coronary obstruction that was treated with ShortCut and Evolut R 23 mm (Medtronic). (*A–C*) Computed tomography measurements showing predicted risk of coronary obstruction. (*D*) Baseline aortogram demonstrating risk for coronary obstruction (side view of left and right coronary cusps). (*E* and *F*) The ShortCut is positioned. Activated splitting element punctures the leaflet from the ventricular side just above the annulus. The leaflet is split by gently retracting the catheter while feeding the guidewire. (*G* and *H*) Echocardiography verifies left and right leaflet split (annotated arrows). (*I*) Post-TAVI implantation and ring fracture with a 23 mm True balloon (Bard) demonstrating normal coronary flow (annotated arrows). Echocardiography post-TAVI (not shown) showed aortic valve gradients of 15/6 mmHg. The patient had an uneventful hospital stay

**Figure 3:**
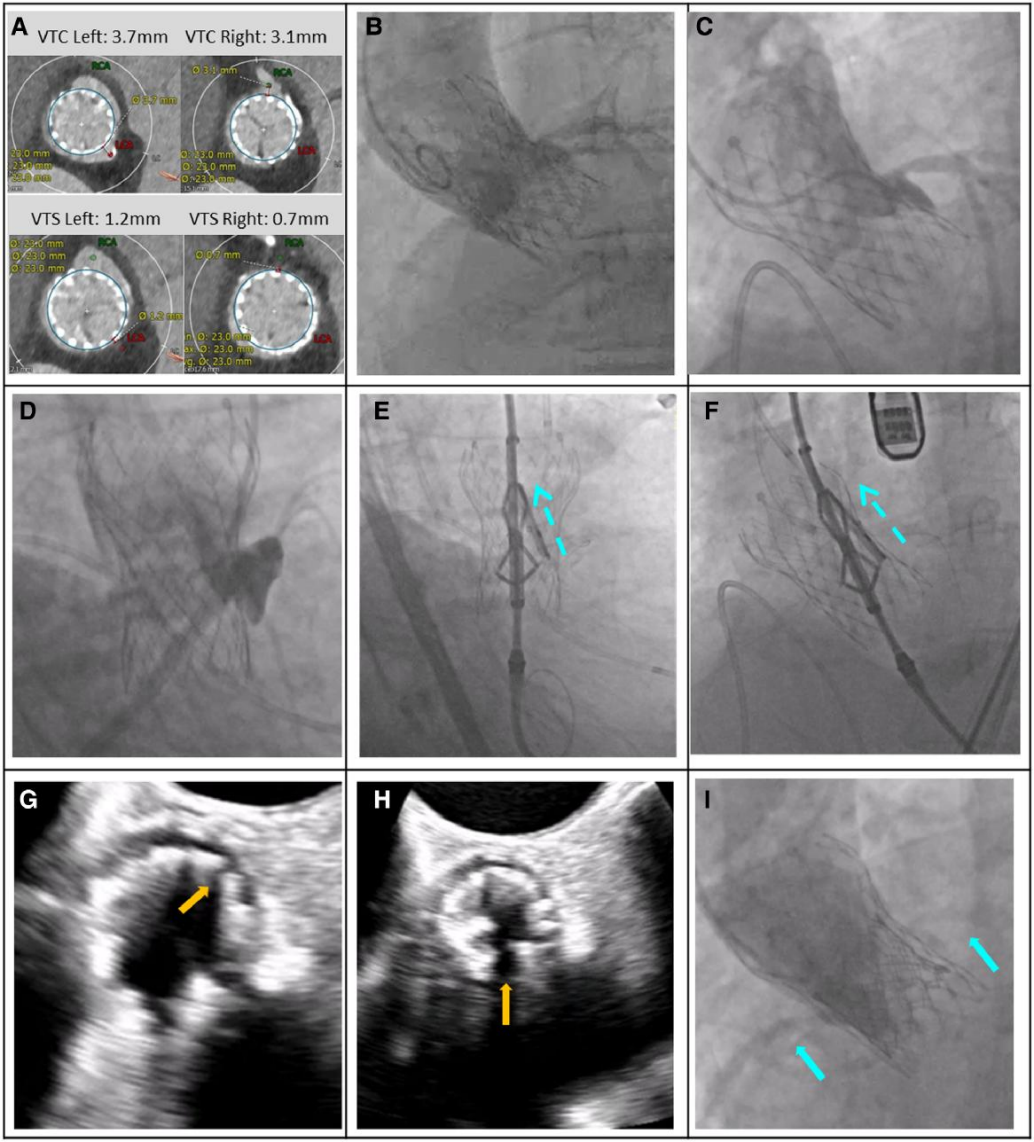
A ShortCut procedure in a patient with a failed transcatheter heart valve. A 79-year-old female with a failed Evolut R 29 mm transcatheter aortic valve (Medtronic) at high risk for double coronary obstruction due to leaflets extending well above the sinotubular junction with effaced sinuses and narrow sinotubular junction, that was treated with ShortCut and was implanted with a 23 mm SAPIEN 3 valve (Edwards Lifesciences). (*A*) Computed tomography measurements showing predicted risk of coronary obstruction. (*B–D*) Baseline aortogram, right and left coronary angiographic demonstrating of the risk for coronary obstruction (side view of left and right coronary cusps). (*E* and *F*) The ShortCut is positioned. Activated splitting element punctures the leaflet from the ventricular side just above the annulus. The leaflet is split by gently retracting the catheter while feeding the guidewire. (*G* and *H*) Echocardiography verifies left and right leaflet split (annotated arrows). (*I*) Post-TAVI demonstration of normal coronary flow (annotated arrows). Echocardiography post-TAVI (not shown) showed aortic valve gradients of 11/5 mmHg. The patient had an uneventful hospital stay

From January 2022 to September 2023, 137 patients were consented, 71 patients were excluded, and 66 eligible patients were enrolled at 22 clinical sites (see Supplementary data online, *Figure S2*). Key exclusions were excessive leaflet calcification (10.2%), unsuitable anatomy for ShortCut (5.8%), prior leaflet tear (5.8%), and no risk for coronary obstruction (5.1%). A total of 60 patients underwent the ShortCut procedure. Six patients dropped out before treatment due to death prior to index procedure (*n* = 2), withdrawal by the principal investigator (*n* = 2), or withdrawal of consent (*n* = 2). A total of 20 centres (90.9%) had no prior ShortCut experience. The median number of patients per site was 2 (interquartile range 1–4). Patient demographics and baseline clinical characteristics are presented in *Table 1*. Mean patient age was 77.0 ± 9.6 years, and most participants were female (70.0%). In 41.7% of cases, the failed bioprosthetic valve was an externally mounted surgical valve [Trifecta (Abbott) or Mitroflow (LivaNova)], while 3.3% of the patients had a failed transcatheter heart valve (see Supplementary data online, *Table S4*). A total of 56.6% of patients had a small failed bioprosthetic valve label size (≤21 mm). All patients (100%) were determined to be at risk for coronary obstruction. Anatomical characteristics included mean VTC distance of 3.3 ± 1.2 mm (70% < 4 mm), mean VTS distance of 2.2 ± 1.4 mm (45% < 2 mm), and coronary height < 10 mm for 91.7% of the patients. Case examples are provided in *Figures 2* and *3*.

**Table 1:** Patient demographics and baseline characteristics

Patients, *n*	60
Age, years	77.0 ± 9.6
Female sex	42 (70.0)
STS score, %	4.5 ± 2.4
Surgical risk (assessed by the heart team)	
Intermediate/low	0
High	54 (90.0)
Extremely high	6 (10.0)
NYHA class III–IV	40 (66.7)
Coronary artery disease	26 (43.3)
Peripheral vascular disease	6 (10.0)
Diabetes mellitus	15 (25.0)
Renal impairment	
Moderate (50 < CrCl < 85 mL/min)	24 (40.0)
Severe (CrCl < 50 mL/min)	20 (33.3)
Chronic lung disease	8 (13.3)
Chronic liver disease	4 (6.7)
Hypertension	51 (85.0)
Prior percutaneous coronary intervention	13 (21.7)
Prior coronary artery bypass grafting	13 (21.7)
Prior stroke	7 (11.7)
Prior myocardial infarction	3 (5.0)
Atrial fibrillation	14 (23.3)
Pacemaker	6 (10.0)
Left ventricular ejection fraction, %	54.2 ± 10.4
AV peak gradient, mmHg	65.3 ± 21.3
AV mean gradient, mmHg	38.1 ± 13.2
AVA, cm^2^	1.0 ± 0.5
≥Moderate aortic regurgitation	19 (32)
Time to reintervention (since bioprosthetic valve implant), years	10.0 ± 3.5
Failed valve type	
SAVR	58 (96.7)
Stented, internally mounted	29 (48.3)
Stented, externally mounted	25 (41.7)
Stentless	4 (6.7)
TAVI	2 (3.3)
Aortic valve disease	
Isolated aortic stenosis (AS)	35 (58.3)
Isolated aortic regurgitation (AR)	7 (11.7)
Mixed failure (AS and AR)	18 (30.0)
Failed bioprosthetic valve label size, mm	22.1 ± 2.4
19 mm	8 (13.3)
21 mm	26 (43.3)
≥23 mm	26 (43.3)
CT characteristics	
Coronary height, mm	6.9 ± 2.7
Coronary height < 10 mm	55 (91.7)
Coronary ostia eccentricity, deg	10.6 ± 8.0
Sinus of Valsalva width, mm	27.9 ± 3.3
Sinus of Valsalva height, mm	16.2 ± 3.2
VTC distance, mm	3.3 ± 1.2
VTS distance, mm	2.2 ± 1.4
Risk of aortic root anatomy for coronary obstruction^a^	
VIVID type I/IIA/IIIA (low risk)	0 (0)
VIVID type IIB (high risk)	35 (43)
VIVID type IIIB (high risk)	32 (39)
VIVID type IIIC (high risk)	15 (18)

Values are *n* (%) or mean ± SD.

AV, aortic valve; AVA, aortic valve area; CrCl, creatinine clearance; LVEF, left ventricular ejection fraction; NYHA, New York Heart Association; PCI, percutaneous coronary intervention; SAVR, surgical aortic valve replacement; STS, Society of Thoracic Surgeons; TAVI, transcatheter aortic valve implantation; CT, computed tomography; VTC, virtual transcatheter heart valve to coronary artery; VTS, virtual transcatheter heart valve to sinotubular junction; VIVID, Valve-in-Valve International Data.

^a^Total number of split aortic valve leaflets (*n* = 82): left (*n* = 30), right (*n* = 8), left and right (*n* = 22 × 2).

### Procedural details

Procedural details are summarized in *Table 2*. Successful leaflet splitting was achieved in all patients on the first attempt. Single leaflet split was performed in 63.3% of patients, and dual leaflet split was performed in 36.7% of patients. Overall, the ShortCut procedure time was 30.6 ± 17.9 min (single split 26.9 ± 19.7 min, dual split 37.0 ± 14.7 min) including split confirmation by TEE and aortography. The increase in aortic regurgitation caused by leaflet splitting was well tolerated in all patients, except for one patient (1.7%) who experienced transient hypotension after dual leaflet split and ring fracture prior to TAVI, which was quickly implanted, and the patient stabilized. In all patients, valves were implanted successfully following the ShortCut procedure.

**Table 2:** Procedural details

Intervened leaflet	
Left	30 (50.0)
Right	8 (13.3)
Left and right	22 (36.7)
ShortCut procedure time including split verification by TEE, min^a^	30.6 ± 17.9
Single split, min	26.9 ± 19.7
Dual split, min	37.0 ± 14.7
Fracture of bioprosthetic valve frame during TAVI	14 (23.3)
Pre-TAVI^b^	6 (10)
Post-TAVI	8 (13.3)
Implanted transcatheter valve	
Balloon expandable	20 (33.3)
Self-expanding	40 (66.7)
Label size, mm	23.5 ± 1.9
Contrast media total, mL	125.6 ± 65.5
Post-TAVI assessments^c^	
>Mild aortic regurgitation	0 (0)
Mean gradient, mmHg	8.8 ± 5.1

Values are *n* (%) or mean ± SD.

TAVI, transcatheter aortic valve replacement; TEE, transoesophageal echocardiography.

^a^ShortCut procedure time: time between ShortCut catheter insertion to its full retrieval including imaging time for split documentation.

^b^Fracture performed post-ShortCut and Pre-TAVI.

^c^Assessed intra-procedurally by echocardiography and angiography.

### Clinical outcomes

Clinical outcomes are summarized in *Table 3*.

**Table 3:** Clinical outcomes

	*n* (%)	95% CI
Primary efficacy endpoint (per-patient leaflet splitting success)	60 (100)	(94.0–100.0)
Freedom from primary safety endpoint^a^	59 (98.3)	(91.1–100.0)
All-cause mortality	0 (0)	(0.0–6.0)
All-cause stroke	1 (1.7)	(0.0–8.9)
Secondary efficacy endpoint (per-leaflet splitting success)^b^	80 (98.8)	(91.8–99.8)
Secondary safety endpoints (30 days post-procedure)		
All-cause mortality	2 (3.3)	(0.4–11.5)
Cardiovascular mortality	0 (0.0)	(0.0–6.0)
All-cause stroke	1 (1.7)	(0.0–8.9)
Fatal stroke	0 (0)	(0.0–6.0)
Stroke with disability	1 (1.7)	(0.0–8.9)
Stroke without disability	0 (0)	(0.0–6.0)
Coronary obstruction	3 (5.0)	(1.0–13.9)
Right coronary	2 (3.3)	(0.4–11.5)
Left coronary	1 (1.7)	(0.0–8.9)
MI with new evidence of coronary artery obstruction requiring intervention	2 (3.3)	(0.4–11.5)
Major vascular complication	0 (0)	(0.0–6.0)
Cardiac tamponade	1 (1.7)	(0.0–8.9)
Acute kidney injury stage 3–4	2 (3.3)	(0.4–11.5)
Access-related type 3–4 bleeding	0 (0)	(0.0–6.0)
Permanent pacemaker	6 (10.0)	(3.8–20.5)
Other endpoints		
Technical success	59 (98.3)	(91.1–100.0)
90 days post-procedure		
All-cause mortality	3 (5.0)	(1.0–13.9)
All-cause stroke	1 (1.7)	(0.0–8.9)
MI with new evidence of coronary artery obstruction requiring intervention	2 (3.3)	(0.4–11.5)

Values are *n* (%) or mean ± SD. Confidence intervals are calculated using Clopper–Pearson score method.

MI, myocardial infarction; AR, aortic regurgitation.

^a^Defined as 7-day or discharge mortality or stroke related to procedure or ShortCut device.

^b^Total number of split valve leaflets (*n* = 82). Imaging to determine evidence of split was performed on 81 leaflets, as imaging for one patient was not available. The rate of per-leaflet splitting success along with its confidence interval was estimated based on generalized estimating equations, with patient random effect to account for potential dependency between the leaflets of the same patient.

### Primary endpoints

The primary efficacy endpoint of evidence for per-patient leaflet splitting success was achieved in all patients (100%; 95% CI 94%–100%, *P* < .001). Direct split visualization on TEE was achieved in 54 patients (90%) and an increase in aortic regurgitation by either TEE or angiography post-split was achieved in 55 patients (91.7%). Freedom from the primary safety endpoints of mortality or stroke related to the procedure or ShortCut device (at 7-day or discharge) was achieved in 59 patients (98.3%; 95% CI 91.1%–100%), with no mortality, and with one patient experiencing a disabling stroke 4 days post-procedure. This patient had a history of bilateral carotid stenosis and previous cerebrovascular events associated with prior TAVI.

### Secondary endpoints

The secondary efficacy endpoint of evidence for per-leaflet split was achieved in 80 out of 81 leaflets (98.8%; one leaflet out of the 82 split leaflets did not have adequate imaging for assessment). Out of all successful leaflet splits, direct split visualization on TEE was achieved in 73 out of 80 leaflets (91.3%). An increase in aortic regurgitation by either TEE or angiography was achieved in 68 out of 80 leaflets (85.0%).

Two (3.3%) patients died within 30 days of the procedure of non-cardiovascular causes (19-day post-procedure, diverticulitis; 24 days post-procedure, sepsis). Two patients (3.3%) with a known history of significant renal impairment required temporary dialysis after the procedure. One patient (1.7%) had cardiac tamponade caused by guidewire position in the left ventricle. No major vascular complications or access-related type 3–4 bleeding occurred.

Freedom from intervened leaflet-related intraprocedural interventions for coronary artery obstruction was observed in 57 patients (95%; 95% CI 86.1%–99.0%). The three obstructions were successfully managed by stenting and in these cases, there was evidence that leaflets were successfully split. All coronary interventions were performed using standard percutaneous coronary intervention technique, with no surgical bailout. All three patients were alive within 90 days of follow-up. None of the obstructions occurred during the ShortCut leaflet splitting procedure, but rather after TAVI. One obstruction was partial and was managed with successful percutaneous coronary stenting of the right coronary artery. The second obstruction was a complete occlusion of the right coronary artery and occurred one day after TAVI (advanced conduction disorder with ischaemia) and was successfully treated percutaneously with a coronary stent. The third obstruction was partial and did not occur immediately after TAVI but rather after a subsequent prolonged post-dilation with a balloon aiming to fracture the frame of a degenerated bioprosthetic valve. That patient had a single remaining vessel (left main) perfusing an occluded right coronary via collateral, with low left ventricular ejection fraction of 30%. While no angiographic left main obstruction was demonstrated post-TAVI or post-balloon dilation, site investigators elected to perform left main stenting due to profound haemodynamic instability.

### Other endpoints

Within 90 days, freedom from mortality was achieved in 57 patients (95%; 95% CI 86.1%–99.0%), with one additional death (non-cardiovascular) that was reported 57 days post-procedure due to non-specific general deterioration.

ShortCut technical success was achieved in 59 patients (98.3%; 95% CI 91.1%–100.0%) with one patient (1.7%) who had the cardiac tamponade. All patients underwent successful ShortCut leaflet splitting and device retrieval.

## Discussion

This pivotal study aimed to assess the safety and efficacy of the ShortCut leaflet splitting device in patients with failed bioprosthetic aortic valves undergoing TAVI and at risk for coronary obstruction. The key findings of this study are as follows: (i) ShortCut achieved successful leaflet split in 100% of patients, including more than one-third of patients with dual split; (ii) the ShortCut device met the safety endpoints, with no procedural mortality and only 1 stroke (1.7%) within 7 days post-procedure or at discharge; (iii) in three patients (5%) requiring intervention for coronary obstruction post-TAVI, ShortCut leaflet split enabled successful coronary access, optimal coronary stenting, and no mortality; and (iv) despite being used for the first time by most of the operators, leaflet split was achieved efficiently with a single attempt in all cases (*Structured Graphical Abstract*).

Among all patients undergoing TAVI, ViV cases represent a population with therapeutic challenges. Indeed, these patients are known to be at higher risk for procedural complications, intrinsic to the fact that they already had an open-heart surgery (or a transcatheter procedure) and may have anatomical features making TAVI more complex. The current study demonstrates favourable outcomes compared with prior retrospective series,^2,4^ with all TAVI cases completed successfully with no procedural mortality. These findings are extremely important since many patients who present with bioprosthetic valve failure and are at risk for coronary obstruction are managed conservatively or sent to surgery. The study demonstrates that leaflet splitting using ShortCut may allow for safe treatment of patients who otherwise would have no viable option. Coronary obstruction post-TAVI is associated with increased risk of mortality; up to 50% in some series.^4^ In contrast, in our 60-patient at-risk cohort treated with ShortCut, there was no cardiovascular mortality. Moreover, the few (5%) cases with compromised coronary flow post-TAVI were treated successfully by coronary intervention, indicating the potential benefit of pre-procedural splitting for future coronary access. While the current proportion of TAV-in–TAV out of the ViV population, as reported in the 2021 TVT Registry,^17^ is <10% as was the case also in the current study, we believe this proportion will increase significantly over the next few years, allowing for further broadening of the experience in additional failed TAVI valves.

Interestingly, women represent 70% of our study population. The higher female representation may be explained by the following: women are known to have smaller surgical/TAVI valves at initial implantation, increasing the risk of valve failure; and the use of certain types of surgical valves with more favourable initial haemodynamic profile (i.e. stentless, externally mounted leaflet) but known to be more challenging to treat with ViV TAVI are more often used in women. Additionally, women have been shown to experience higher risk of vascular and bleeding complications compared with men when undergoing TAVI. While the size of our study was insufficient to determine gender-specific conclusions, it is reassuring that ShortCut was associated with favourable outcomes among women, reinforcing the safety and efficacy of the study device and procedure.

To date, two distinct techniques are used to minimize the risk of TAVI-induced coronary obstruction in patients considered at risk for this complication. Chimney stenting involves placement of a long coronary stent from the at-risk coronary ostium into the aorta between the target leaflet and the aortic wall to preserve coronary flow.^11^ While the technique is familiar to most interventional cardiologists and has been proven effective in retrospective observational studies,^9–11^ it is limited by not dealing with the original pathology (e.g. the deflected leaflet of the failed valve), in addition to the possibility of stent deformation, stent failure, challenging future coronary access, and the need for prolonged dual antiplatelet therapy.

The BASILICA technique is another option for leaflet splitting that has been reported by several registries.^7,18–21^ Multiple percutaneous and surgical electrocautery tools are used off-label to position a wire, traverse the leaflet, and then snare the wire on the ventricular side to lacerate the leaflet. These potentially time-consuming positioning steps have thus far limited BASILICA use to highly experienced operators, resulting in underutilization of leaflet modification, despite its recognized benefits.

ShortCut is fundamentally different from BASILICA by design: its dedicated catheter system allows for easy stabilization and visualization of the splitting elements under fluoroscopy, with an intuitive ability to centre, orient and accurately determine the splitting trajectory and without the need for leaflet traversal by a wire or snaring. As a result, training new operators to perform ShortCut is straightforward, as evidenced in the current study by short procedure times for centres without prior device experience. Moreover, the ShortCut is delivered over the TAVI wire, with continuous access to the ventricle and prompt valve delivery after leaflet splitting, while in BASILICA procedures double aortic valve crossing is required. Finally, dual leaflet splitting could be performed promptly with one ShortCut device, while BASILICA requires two distinct setups. While our study was not designed to compare different splitting approaches, the results indicate that ShortCut not only met its safety and efficacy endpoints, but is also easy to teach and use. It therefore allows at risk ViV procedures to be performed without disrupting the cathlab workflow.

This study has several important limitations. First, it was a non-randomized study of modest size that did not compare different methods to address the risk of coronary obstruction. Due to the high mortality and morbidity associated with coronary obstruction post-TAVI, a randomized trial comparing leaflet splitting with ShortCut with the current standard of care (no protection or off-label use of devices or techniques) will be challenging to conduct and potentially unethical. Second, the use of TEE and cerebral protection devices was a derivative of the study design but may not be required in real-life practice. Additional imaging modalities, such as intravascular ultrasound, could be useful for further assessment of coronary artery patency, but were not part of the study protocol.^22^ Third, the study treatment group was ∼50% of the screened patient cohort. Exclusions related to excessive leaflet calcification (10.2%) and unsuitable anatomy for ShortCut (5.8%) are expected to be reduced further with physician experience and device iterations. Finally, this study focused on leaflet splitting in patients with failed bioprosthetic aortic valves. Future experience will be needed to explore the use of ShortCut in native aortic valves as well as in mitral valves, and with additional failed TAVI devices.

In conclusion, modification of failed bioprosthetic aortic valve leaflets using ShortCut was safe, achieved successful leaflet splitting in all patients, and was associated with favourable clinical outcomes in patients at risk for coronary obstruction undergoing TAVI.

## Supplementary data

Supplementary data are available at *European Heart Journal* online.

## Supplementary Material

ehae303_Supplementary_Data

## Declarations

### Data Availability

The data underlying this article are available in the article and in its online supplementary material.

### Pre-registered Clinical Trial Number

NCT04952909.
