# Supplementary material for: Leaflet modification before transcatheter aortic valve implantation in patients at risk for coronary obstruction: the ShortCut study
Source: Eur Heart J. 2024 May 15;45(33):3031–41. doi: 10.1093/eurheartj/ehae303 (PMC11365606; doi:10.1093/eurheartj/ehae303)
Supplement: ehae303_Supplementary_Data [file ehae303_supplementary_data.docx]

**Supplemental Material**

**Leaflet Modification Before Transcatheter Aortic Valve Implantation**

**In Patients At Risk For Coronary Obstruction: The ShortCut Study**

Danny Dvir, MD, et al.

**Supplementary Table 1. ShortCut Pivotal Study – List of Principal Investigators**

| **Site** | **Principal Investigator** |
| --- | --- |
| **Advocate Christ Medical Center** Oak Lawn, Illinois | Ravi K. Ramana |
| **Baylor Scott & White Research Institute** Plano, Texas | Molly Szerlip |
| **Carolinas Medical Center**  Charlotte, North Carolina | Michael Rinaldi |
| **Cedars-Sinai Medical Center** Los Angeles, California | Raj Makkar |
| **CHU de Bordeaux** Bordeaux, France | Benjamin Seguy |
| **Clinique Pasteur Toulouse** Toulouse, France | Didier Tchétché (**Global Study PI**) |
| **Columbia University Medical Center** New York, New York | Tamim Nazif  Susheel K. Kodali (**Global Study PI**, not site PI) |
| **German Heart Institute Berlin Charité** Berlin, Germany | Jörg Kempfert |
| **Institute Mutualiste Montsouris** Paris, France | Christophe Caussin |
| **Intermountain Medical Center** Murray, Utah | Brian Whisenant |
| **Kaiser Permanente Research Institute S**an Francisco, California | Jacob Mishell |
| **Leeds General Infirmary** Leeds, UK | Daniel Blackman |
| **Leipzig Heart Institute GmbH** Leipzig, Germany | Mohammed Abdel-Wahab |
| **Los Robles Medical Center** Thousand Oaks, California | Saibal Kar |
| **Medstar Washington Hospital Center** Washington, DC | Itzik Ben-Dor |
| **Morristown Medical Center,** Morristown, New Jersey | Philippe Généreux |
| **Rabin Medical Center** Petah Tikva, Israel | Ran Kornowski |
| **Royal Sussex County Hospital** Brighton, UK | David Hildick-Smith |
| **Sha'are Tzedek Medical Center** Jerusalem, Israel | Danny Dvir (**Global Study PI**) |
| **Tucson Medical Center** Tucson, Arizona | Thomas Waggoner |
| **University Hospital Hamburg** Hamburg, Germany | Lenard Conradi |
| **University of Michigan,**  Ann Arbor, Michigan | Stanley Chetcuti |
| **UPMC Pinnacle Harrisburg** Wormleysburg, Pennsylvania | Hemal Gada |

A total of 22 out of 23 centers screened ShortCut patients.

**Supplementary Table 2. Frequency Distribution of Risk Factors For Coronary Obstruction After Valve-In-Valve Implantation**

| **Risk Factors** | **N (%)** |
| --- | --- |
| **Anatomical factors** |  |
| Leaflets extension above coronary ostium | 42 (70.0) |
| Short virtual transcatheter heart valve to coronary (VTC) distance | 37 (61.7) |
| Short virtual transcatheter heart valve to sinotubular junction (VTS) distance | 27 (45.0) |
| STJ-leaflet mismatch (Short STJ height minus leaflet length) | 16 (26.7) |
| Low-lying coronary ostia | 49 (81.7) |
| Narrow sinuses of Valsalva | 18 (30.0) |
| Previous root repair (e.g., root graft and coronary reimplantation) | 2 (3.3) |
| **Bioprosthetic valve factors** |  |
| Supra-annular position | 16 (26.7) |
| Internal stent frame (*e.g.*, Mitroflow, Trifecta) | 23 (38.3) |
| No stent frame (homograft, stentless valves) | 4 (6.7) |
| Long, thick or bulky bioprosthetic leaflets | 11 (18.3) |
| Stents posts extension above sinotubular junction | 17 (28.3) |
| Intended over-expansion/fracture of bioprosthetic valve frame during TAVI | 17 (28.3) |
| **Transcatheter valve factors** |  |
| Extended sealing cuff | 19 (31.7) |
| High implantation | 20 (33.3) |

Each subject could have either anatomical risk factor, bioprosthetic valve risk factors or transcatheter valve Risk Factors. One of the risk factors was sufficient for risk of obstruction evaluation.

**Supplementary Table 3. List of Key Exclusion Criteria**

| 1. Patient is not at risk for TAVI-induced coronary artery ostium obstruction. 2. Patient is planned to undergo a percutaneous valve-in-valve procedure for a non-approved ViV indication due to a failed bioprosthetic valve. 3. An excessive aortic valve leaflet calcium morphology, such as diffuse massive calcification at the targeted leaflet for splitting. 4. Anatomy not suitable for the use of the ShortCut™ device. 5. Leaflet planned to be intervened is torn pre-ShortCut™ device access. 6. Patient has iliofemoral vessel characteristics that preclude safe insertion of the introducer sheath. 7. Planned concurrent intervention in the same setting of the index procedure. 8. Surgery or interventional procedure ≤ 1 month prior to the index procedure. 9. Planned provisional (pre-position coronary artery) stents. 10. Coronary disease that, in the opinion of the local Heart Team, should be treated; or treatment of coronary disease ≤ 1 month prior to index procedure. 11. Carotid or vertebral artery disease that, in the opinion of the local Heart Team, should be treated; or treatment of carotid stenosis ≤ 1 month prior to index procedure. 12. CVA or TIA ≤ 6 months prior to index procedure. 13. Severe neurological disability, as determined by the Investigator. 14. History of a myocardial infarction ≤ 6 weeks prior to index procedure. 15. Current or suspected endocarditis on the aortic valve. 16. Identified thrombotic material on the valve by either CT or Echocardiography. 17. Cardiac imaging evidence of left ventricular intracardiac mass or thrombus. 18. Hemodynamic or respiratory instability requiring inotropic support, mechanical ventilation or mechanical heart assistance. 19. LVEF < 30%. 20. Ongoing severe infection or sepsis. 21. Patient refuses blood transfusion. 22. Patient has renal insufficiency (GFR < 30 ml/min or serum creatinine > 2.5 mg/dL), or on chronic dialysis. 23. Patient with clinically significant abnormality in cell blood count as defined by WBC < 3000 cell/μL, Hb < 9 g/dL and platelet count < 90,000 cell/μL or history of bleeding diathesis or coagulopathy, or hypercoagulable states. 24. Anatomy that does not allow safe placement of a cerebral embolic protection device. 25. Active peptic ulcer with bleeding. 26. Known allergy to contrast media that cannot be adequately controlled with premedication. 27. Known hypersensitivity or contraindication to all intra-procedural anticoagulation or any product material. 28. Need for emergency surgery for any reason. 29. Inoperable for emergency open‐heart surgery. 30. Patient has a condition that, in the opinion of the Investigator or the Screening Committee, precludes the patient from undergoing the index procedure. 31. Life expectancy is less than one year. |
| --- |

**Supplementary Table 4. Failed Bioprosthetic Valves Included in the ShortCut Study**

| **Failed Bioprosthetic Valves (N=60)** | **N (%)** |
| --- | --- |
| Magna/Magna Ease/Perimount/Carpentier (Edwards Lifesciences) | 24 (40.0%) |
| Trifecta (Abbott) | 14 (23.3%) |
| Mitroflow/Sorin Crown (Livanova) | 11 (18.3%) |
| Mosaic/Hancock (Medtronic) | 3 (5.0%) |
| Perceval (Livanova) | 2 (3.3%) |
| Evolut (Medtronic) | 2 (3.3%) |
| 3F (Medtronic) | 2 (3.3%) |
| Freedom Solo (Livanova) | 1 (1.7%) |
| Toronto SPV (Abbott) | 1 (1.7%) |

**Supplementary Figure 1. VIVID Classification Type**


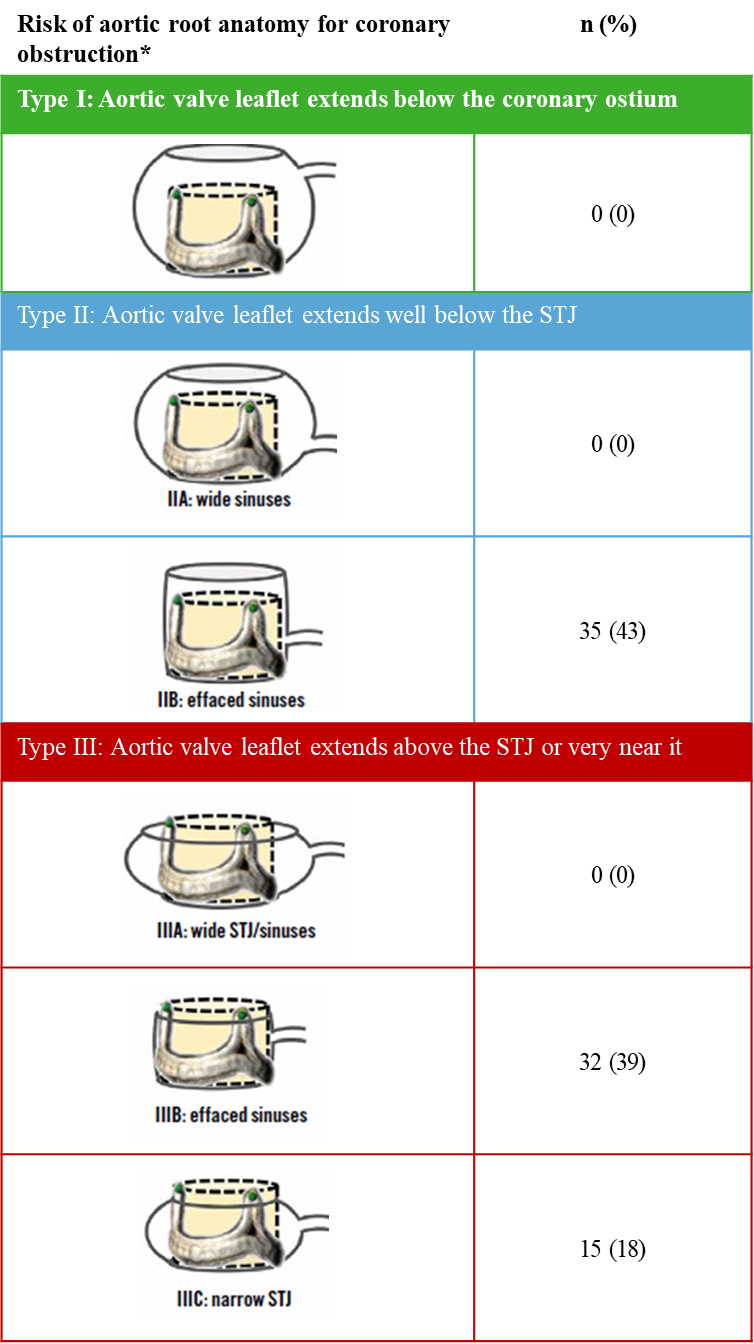


Tang G, et al. VIVID classification. EuroIntervention. 2020 Oct 9;16(9):e757-e759.

The presented information related to the VIVID Classification was performed retrospectively for each patient. The VIVID classification was not part of the patient screening process since this classification requires further clinical verification.

**Supplementary Figure 2. ShortCut Study Flow Chart**

**
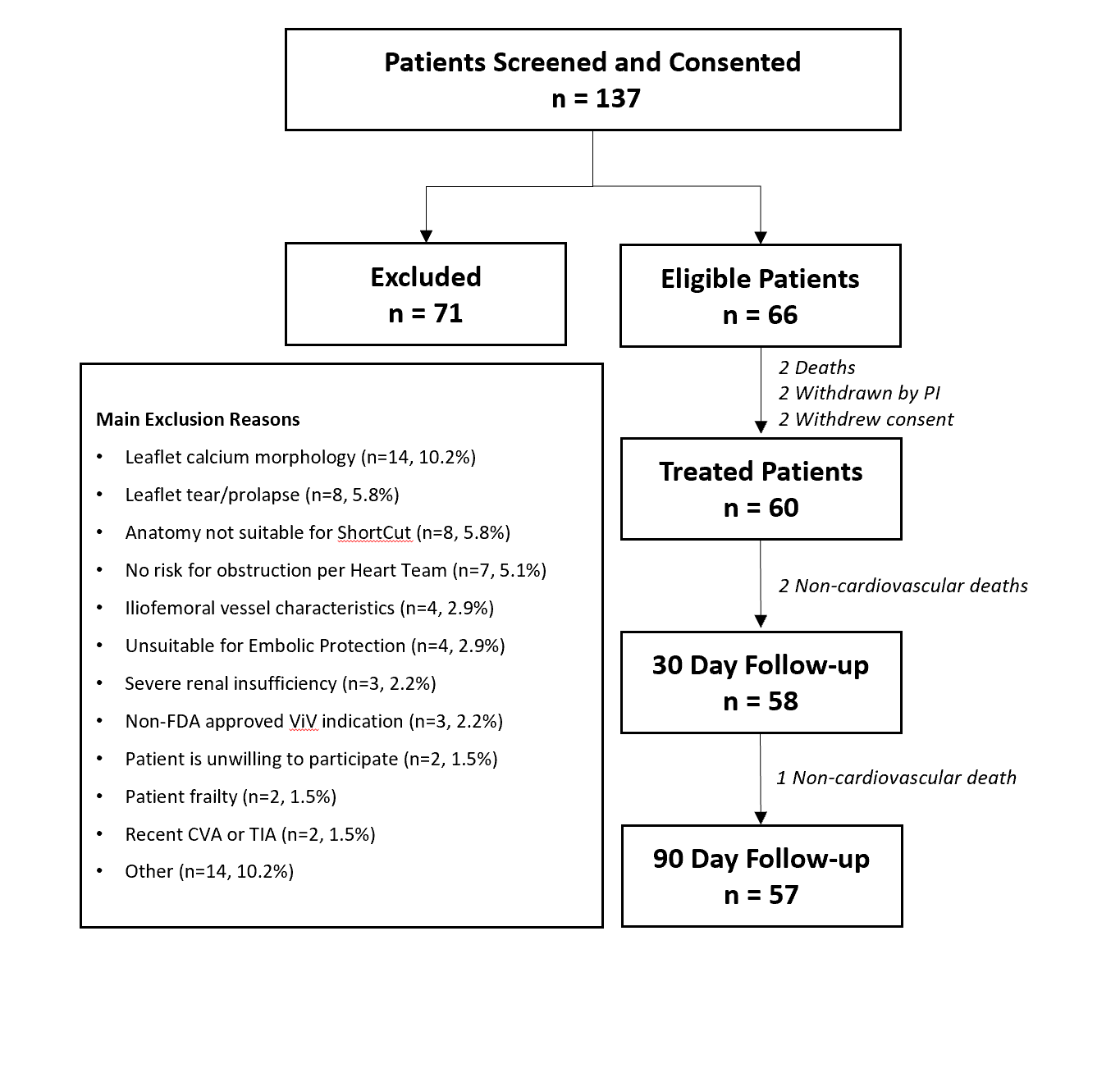
**
